# Prevention and Control of Seasonal Influenza with Vaccines: Recommendations of the Advisory Committee on Immunization Practices — United States, 2024–25 Influenza Season

**DOI:** 10.15585/mmwr.rr7305a1

**Published:** 2024-08-29

**Authors:** Lisa A. Grohskopf, Jill M. Ferdinands, Lenee H. Blanton, Karen R. Broder, Jamie Loehr

**Affiliations:** ^1^Influenza Division, National Center for Immunization and Respiratory Diseases, CDC; ^2^Immunization Safety Office, National Center for Emerging and Zoonotic Infectious Diseases, CDC; ^3^Jamie Loehr, MD, Cayuga Family Medicine, Ithaca, New York

## Abstract

This report updates the 2023–24 recommendations of the Advisory Committee on Immunization Practices (ACIP) concerning the use of seasonal influenza vaccines in the United States (*MMWR Recomm Rep 2022;72[No. RR-2]:1–24*). Routine annual influenza vaccination is recommended for all persons aged ≥6 months who do not have contraindications. Trivalent inactivated influenza vaccines (IIV3s), trivalent recombinant influenza vaccine (RIV3), and trivalent live attenuated influenza vaccine (LAIV3) are expected to be available. All persons should receive an age-appropriate influenza vaccine (i.e., one approved for their age), with the exception that solid organ transplant recipients aged 18 through 64 years who are receiving immunosuppressive medication regimens may receive either high-dose inactivated influenza vaccine (HD-IIV3) or adjuvanted inactivated influenza vaccine (aIIV3) as acceptable options (without a preference over other age-appropriate IIV3s or RIV3). Except for vaccination for adults aged ≥65 years, ACIP makes no preferential recommendation for a specific vaccine when more than one licensed and recommended vaccine is available. ACIP recommends that adults aged ≥65 years preferentially receive any one of the following higher dose or adjuvanted influenza vaccines: trivalent high-dose inactivated influenza vaccine (HD-IIV3), trivalent recombinant influenza vaccine (RIV3), or trivalent adjuvanted inactivated influenza vaccine (aIIV3). If none of these three vaccines is available at an opportunity for vaccine administration, then any other age-appropriate influenza vaccine should be used.

Primary updates to this report include the following two topics: the composition of 2024–25 U.S. seasonal influenza vaccines and updated recommendations for vaccination of adult solid organ transplant recipients. First, following a period of no confirmed detections of wild-type influenza B/Yamagata lineage viruses in global surveillance since March 2020, 2024–25 U.S. influenza vaccines will not include an influenza B/Yamagata component. All influenza vaccines available in the United States during the 2024–25 season will be trivalent vaccines containing hemagglutinin derived from 1) an influenza A/Victoria/4897/2022 (H1N1)pdm09-like virus (for egg-based vaccines) or an influenza A/Wisconsin/67/2022 (H1N1)pdm09-like virus (for cell culture-based and recombinant vaccines); 2) an influenza A/Thailand/8/2022 (H3N2)-like virus (for egg-based vaccines) or an influenza A/Massachusetts/18/2022 (H3N2)-like virus (for cell culture-based and recombinant vaccines); and 3) an influenza B/Austria/1359417/2021 (Victoria lineage)-like virus. Second, recommendations for vaccination of adult solid organ transplant recipients have been updated to include HD-IIV3 and aIIV3 as acceptable options for solid organ transplant recipients aged 18 through 64 years who are receiving immunosuppressive medication regimens (without a preference over other age-appropriate IIV3s or RIV3).

This report focuses on recommendations for the use of vaccines for the prevention and control of seasonal influenza during the 2024–25 influenza season in the United States. A brief summary of the recommendations and a link to the most recent Background Document containing additional information are available at https://www.cdc.gov/acip-recs/hcp/vaccine-specific/flu.html?CDC_AAref_Val=https://www.cdc.gov/vaccines/hcp/acip-recs/vacc-specific/flu.html. These recommendations apply to U.S.-licensed influenza vaccines. Updates and other information are available from CDC’s influenza website (https://www.cdc.gov/flu). Vaccination and health care providers should check this site periodically for additional information.

## Introduction

Influenza viruses typically circulate annually in the United States, most commonly from the late fall through the early spring. Most persons who become ill after influenza virus infection recover without serious complications or sequelae. However, influenza can be associated with serious illnesses, hospitalizations, and deaths, particularly among older adults, very young children, pregnant persons, and persons of all ages with certain chronic medical conditions (*1*–*7*). Influenza also is an important cause of missed work and school (*8*–*10*).

Routine annual influenza vaccination for all persons aged ≥6 months who do not have contraindications has been recommended by CDC and the Advisory Committee on Immunization Practices (ACIP) since 2010 (*11*). Vaccination provides important protection from influenza illness and its potential complications. The effectiveness of influenza vaccination varies depending on multiple factors such as the age and health of the recipient, the type of vaccine administered, the types and subtypes of influenza viruses circulating in the community, and the degree of similarity between circulating viruses and those included in the vaccine (*12*). During each of the six influenza seasons from 2010–11 through 2015–16, influenza vaccination prevented an estimated 1.6–6.7 million illnesses, 790,000–3.1 million outpatient medical visits, 39,000–87,000 hospitalizations, and 3,000–10,000 respiratory and circulatory deaths each season in the United States (*13*). During the severe 2017–18 season, notable for an unusually long duration of widespread high influenza activity throughout the United States and higher rates of outpatient visits and hospitalizations compared with recent seasons, vaccination prevented an estimated 7.1 million illnesses, 3.7 million medical visits, 109,000 hospitalizations, and 8,000 deaths (*14*), despite an overall estimated vaccine effectiveness of 38% (62% against influenza A[H1N1]pdm09 viruses, 22% against influenza A[H3N2] viruses, and 50% against influenza B viruses) (*14*).

This report updates the 2023–24 ACIP recommendations regarding the use of seasonal influenza vaccines (*15*) and provides recommendations and guidance for vaccination providers regarding the use of influenza vaccines in the United States for the 2024–25 season. Various formulations of influenza vaccines are available (Table 1). Contraindications and precautions for the use of influenza vaccines are summarized (Tables 2 and 3). Abbreviations are used in this report to denote the various types of vaccines (Box). A summary of these recommendations and a Background Document containing additional information on influenza, influenza-associated illness, and influenza vaccines are available at https://www.cdc.gov/acip-recs/hcp/vaccine-specific/flu.html?CDC_AAref_Val=https://www.cdc.gov/vaccines/hcp/acip-recs/vacc-specific/flu.html.

**TABLE 1:** Influenza vaccines — United States, 2024–25 influenza season*

Trade name (manufacturer)	Presentations	Age indication	*μ*g HA (IIV3s and RIV3) or virus count (LAIV3) for each vaccine virus (per dose)	Route	Mercury (from thimerosal, if present), *μ*g/0.5 mL
**IIV3s (standard-dose, egg-based vaccines^†^)**					
Afluria (Seqirus)	0.5-mL PFS^§^	≥3 yrs^§^	15 *μ*g/0.5 mL	IM^¶^	—**
	5.0-mL MDV^§^	≥6 mos^§^ (needle and syringe) 18 through 64 yrs (jet injector)	7.5 *μ*g/0.25 mL 15 *μ*g/0.5 mL	IM^¶^	24.5
Fluarix (GlaxoSmithKline)	0.5-mL PFS	≥6 mos	15 *μ*g/0.5 mL	IM^¶^	—
FluLaval (GlaxoSmithKline)	0.5-mL PFS	≥6 mos	15 *μ*g/0.5 mL	IM^¶^	—
Fluzone (Sanofi Pasteur)	0.5-mL PFS^††^	≥6 mos^††^	15 *μ*g/0.5 mL	IM^¶^	—
	5.0-mL MDV^††^	≥6 mos^††^	7.5 *μ*g/0.25 mL 15 *μ*g/0.5 mL	IM^¶^	25
**ccIIV3 (standard-dose, cell culture-based vaccine)**					
Flucelvax (Seqirus)	0.5-mL PFS	≥6 mos	15 *μ*g/0.5 mL	IM^¶^	—
	5.0-mL MDV	≥6 mos	15 *μ*g/0.5 mL	IM^¶^	25
**HD-IIV3 (high-dose, egg-based vaccine^†^)**					
Fluzone High-Dose (Sanofi Pasteur)	0.5-mL PFS	≥65 yrs	60 *μ*g/0.5 mL	IM^¶^	—
**aIIV3 (standard-dose, egg-based vaccine^†^ with MF59 adjuvant)**					
Fluad (Seqirus)	0.5-mL PFS	≥65 yrs	15 *μ*g/0.5 mL	IM^¶^	—
**RIV3 (recombinant HA vaccine)**					
Flublok (Sanofi Pasteur)	0.5-mL PFS	≥18 yrs	45 *μ*g/0.5 mL	IM^¶^	—
**LAIV3 (egg-based vaccine^†^)**					
FluMist (AstraZeneca)	0.2-mL prefilled single-use intranasal sprayer	2 through 49 yrs	10^6.5–7.5^ fluorescent focus units/0.2 mL	NAS	—

**Abbreviations:** ACIP = Advisory Committee on Immunization Practices; aIIV3 = adjuvanted inactivated influenza vaccine, trivalent; ccIIV3 = cell culture-based inactivated influenza vaccine, trivalent; HA = hemagglutinin; HD-IIV3 = high-dose inactivated influenza vaccine, trivalent; IIV3 = inactivated influenza vaccine, trivalent; IM = intramuscular; LAIV3 = live attenuated influenza vaccine, trivalent; MDV = multidose vial; NAS = intranasal; PFS = prefilled syringe; RIV3 = recombinant influenza vaccine, trivalent.

* Manufacturer package inserts and updated CDC and ACIP guidance should be consulted for additional information concerning, but not limited to, indications, contraindications, warnings, and precautions. Package inserts for U.S.-licensed vaccines are available at https://www.fda.gov/vaccines-blood-biologics/vaccines/vaccines-licensed-use-united-states. Availability and characteristics of specific products and presentations might change or differ from what is described in this table and in the text of this report.

^†^ Although a history of severe allergic reaction (e.g., anaphylaxis) to egg is a labeled contraindication to the use of egg-based IIV3s and LAIV3, ACIP recommends that all persons aged ≥6 months with egg allergy should receive influenza vaccine and that any influenza vaccine (egg based or nonegg based) that is otherwise appropriate for the recipient’s age and health status can be used (see Persons with a History of Egg Allergy).

^§^ The approved dose volume for Afluria is 0.25 mL for children aged 6 through 35 months and 0.5 mL for persons aged ≥3 years. However, 0.25-mL prefilled syringes are no longer available. For children aged 6 through 35 months, a 0.25-mL dose must be obtained from a multidose vial.

^¶^ IM-administered influenza vaccines should be administered by needle and syringe only, except for the MDV presentation of Afluria, which can alternatively be given by the PharmaJet Stratis jet injector for persons aged 18 through 64 years only. For older children and adults, the recommended site for IM influenza vaccination is the deltoid muscle. The preferred site for infants and young children is the anterolateral aspect of the thigh. Additional specific guidance regarding site selection and needle length for IM administration is available in the General Best Practice Guidelines for Immunization available at https://www.cdc.gov/vaccines/hcp/acip-recs/general-recs/index.html.

** Not applicable.

^††^ Fluzone is approved for children aged 6 through 35 months at either 0.25 mL or 0.5 mL per dose; however, 0.25-mL prefilled syringes are no longer available. If a prefilled syringe of Fluzone is used for a child in this age group, the dose volume will be 0.5 mL per dose.

**TABLE 2:** Contraindications and precautions for the use of influenza vaccines — United States, 2024–25 influenza season*

Vaccine type	Contraindications	Precautions
**Egg-based IIV3s**	History of severe allergic reaction (e.g., anaphylaxis) to any component of the vaccine^†^ or to a previous dose of any influenza vaccine (i.e., any egg-based IIV, ccIIV, RIV, or LAIV)^§^	Moderate or severe acute illness with or without fever History of Guillain-Barré syndrome within 6 weeks of receipt of influenza vaccine
**ccIIV3**	History of severe allergic reaction (e.g., anaphylaxis) to a previous dose of any ccIIV or any component of ccIIV3^§^	Moderate or severe acute illness with or without fever History of Guillain-Barré syndrome within 6 weeks of receipt of influenza vaccine History of severe allergic reaction to a previous dose of any other influenza vaccine (i.e., any egg-based IIV, RIV, or LAIV)^¶^
**RIV3**	History of severe allergic reaction (e.g., anaphylaxis) to a previous dose of any RIV or any component of RIV3^§^	Moderate or severe acute illness with or without fever History of Guillain-Barré syndrome within 6 weeks of receipt of influenza vaccine History of severe allergic reaction to a previous dose of any other influenza vaccine (i.e., any egg-based IIV, ccIIV, or LAIV)^¶^
**LAIV3**	History of severe allergic reaction (e.g., anaphylaxis) to any component of the vaccine^†^ or to a previous dose of any influenza vaccine (i.e., any egg-based IIV, ccIIV, RIV, or LAIV)^§^ Concomitant aspirin- or salicylate-containing therapy in children and adolescents^§^ Children aged 2 through 4 years who have received a diagnosis of asthma or whose parents or caregivers report that a health care provider has told them during the preceding 12 months that their child had wheezing or asthma or whose medical record indicates a wheezing episode has occurred during the preceding 12 months Children and adults who are immunocompromised due to any cause, including but not limited to immunosuppression caused by medications, congenital or acquired immunodeficiency states, HIV infection, anatomic asplenia, or functional asplenia (e.g., due to sickle cell anemia) Close contacts and caregivers of severely immunosuppressed persons who require a protected environment Pregnancy Persons with active communication between the CSF and the oropharynx, nasopharynx, nose, or ear or any other cranial CSF leak Persons with cochlear implants** Receipt of influenza antiviral medication within the previous 48 hours for oseltamivir and zanamivir, previous 5 days for peramivir, and previous 17 days for baloxavir^††^	Moderate or severe acute illness with or without fever History of Guillain-Barré syndrome within 6 weeks of receipt of influenza vaccine Asthma in persons aged ≥5 years Other underlying medical conditions that might predispose to complications after wild-type influenza infection (e.g., chronic pulmonary, cardiovascular [except isolated hypertension], renal, hepatic, neurologic, hematologic, or metabolic disorders [including diabetes mellitus])

**Abbreviations:** ACIP = Advisory Committee on Immunization Practices; ccIIV = cell culture–based inactivated influenza vaccine (any valency); ccIIV3 = cell culture–based inactivated influenza vaccine, trivalent; CSF = cerebrospinal fluid; IIV = inactivated influenza vaccine (any valency); IIV3 = inactivated influenza vaccine, trivalent; LAIV = live attenuated influenza vaccine (any valency); LAIV3 = live attenuated influenza vaccine, trivalent; RIV = recombinant influenza vaccine (any valency); RIV3 = recombinant influenza vaccine, trivalent.

* Manufacturer package inserts and updated CDC and ACIP guidance should be consulted for additional information concerning, but not limited to, indications, contraindications, warnings, and precautions. When a contraindication is present, a vaccine should not be administered. When a precaution is present, vaccination should generally be deferred but might be indicated if the benefit of protection from the vaccine outweighs the risk for an adverse reaction (see the General Best Practice Guidelines for Immunization, available at https://www.cdc.gov/vaccines/hcp/acip-recs/general-recs/index.html). Package inserts for U.S.-licensed vaccines are available at https://www.fda.gov/vaccines-blood-biologics/vaccines/vaccines-licensed-use-united-states.

^†^ Although a history of severe allergic reaction (e.g., anaphylaxis) to egg is a labeled contraindication to the use of egg-based IIV3s and LAIV3, ACIP recommends that all persons aged ≥6 months with egg allergy should receive influenza vaccine, and that any influenza vaccine (egg based or nonegg based) that is otherwise appropriate for the recipient’s age and health status can be used (see Persons with a History of Egg Allergy).

^§^ Labeled contraindication noted in package insert.

^¶^ If administered, vaccination should occur in a medical setting and should be supervised by a health care provider who can recognize and manage severe allergic reactions. Providers can consider consultation with an allergist in such cases to assist in identification of the component responsible for the allergic reaction.

** Injectable vaccines are recommended for persons with cochlear implant because of the potential for CSF leak, which might exist for a period after implantation. Providers might consider consultation with a specialist concerning risk for persistent CSF leak if an inactivated or recombinant vaccine cannot be used.

^††^ Use of LAIV3 in context of influenza antivirals has not been studied; however, interference with activity of LAIV3 is biologically plausible, and this possibility is noted in the package insert for LAIV3. In the absence of data supporting an adequate minimum interval between influenza antiviral use and LAIV3 administration, the intervals provided are based on the half-life of each antiviral. The interval between influenza antiviral receipt and LAIV3 for which interference might potentially occur might be further prolonged in the presence of medical conditions that delay medication clearance (e.g., renal insufficiency). Influenza antivirals might also interfere with LAIV3 if initiated within 2 weeks after vaccination. Persons who receive antivirals during the period starting with the specified time before receipt of LAIV3 through 2 weeks after receipt of LAIV3 should be revaccinated with an age-appropriate IIV3 or RIV3.

**TABLE 3:** Influenza vaccine contraindications and precautions for persons with a history of severe allergic reaction to a previous dose of influenza vaccine* — United States, 2024–25 influenza season

Vaccine (of any valency) associated with previous severe allergic reaction (e.g., anaphylaxis)	Available 2024–25 influenza vaccines		
	Egg based IIV3s and LAIV3	ccIIV3	RIV3
Any egg based IIV or LAIV	Contraindication^†^	Precaution^§^	Precaution^§^
Any ccIIV	Contraindication^†^	Contraindication^†^	Precaution^§^
Any RIV	Contraindication^†^	Precaution^§^	Contraindication^†^
Unknown influenza vaccine	Allergist consultation recommended		

**Abbreviations:** ACIP = Advisory Committee on Immunization Practices; ccIIV = cell culture–based inactivated influenza vaccine (any valency); ccIIV3 = cell culture–based inactivated influenza vaccine, trivalent; IIV = inactivated influenza vaccine (any valency); IIV3 = inactivated influenza vaccine, trivalent; LAIV = live attenuated influenza vaccine (any valency); LAIV3 = live attenuated influenza vaccine, trivalent; RIV = recombinant influenza vaccine (any valency); RIV3 = recombinant influenza vaccine, trivalent.

* Manufacturer package inserts and updated CDC and ACIP guidance should be consulted for additional information, including, but not limited to indications, contraindications, warnings, and precautions. Package inserts for U.S.-licensed vaccines are available at https://www.fda.gov/vaccines-blood-biologics/vaccines/vaccines-licensed-use-united-states.

^†^ When a contraindication is present, a vaccine should not be administered, consistent with the General Best Practice Guidelines for Immunization (Source: Kroger A, Bahta L, Long S, Sanchez P. General best practice guidelines for immunization; https://www.cdc.gov/vaccines/hcp/acip-recs/general-recs/index.html). In addition to the contraindications based on history of severe allergic reaction to influenza vaccines that are noted in the table, each individual influenza vaccine is contraindicated for persons who have had a severe allergic reaction (e.g., anaphylaxis) to any component of that vaccine. Vaccine components can be found in package inserts. Although a history of severe allergic reaction (e.g., anaphylaxis) to egg is a labeled contraindication to the use of egg-based IIV3s and LAIV3, ACIP recommends that all persons aged ≥6 months with egg allergy should receive influenza vaccine, and that any influenza vaccine (egg based or nonegg based) that is otherwise appropriate for the recipient’s age and health status can be used (see Persons with a History of Egg Allergy).

^§^ When a precaution is present, vaccination should generally be deferred but might be indicated if the benefit of protection from the vaccine outweighs the risk for an adverse reaction, consistent with the General Best Practice Guidelines for Immunization (Source: Kroger A, Bahta L, Long S, Sanchez P. General best practice guidelines for immunization; https://www.cdc.gov/vaccines/hcp/acip-recs/general-recs/index.html). Providers can consider using the following vaccines in these instances; however, vaccination should occur in an inpatient or outpatient medical setting with supervision by a health care provider who is able to recognize and manage severe allergic reactions: 1) for persons with a history of severe allergic reaction (e.g., anaphylaxis) to any egg-based IIV or LAIV of any valency, the provider can consider administering ccIIV3 or RIV3; 2) for persons with a history of severe allergic reaction (e.g., anaphylaxis) to any ccIIV of any valency, the provider can consider administering RIV3; and 3) for persons with a history of severe allergic reaction (e.g., anaphylaxis) to any RIV of any valency, the provider can consider administering ccIIV3. Providers can also consider consulting with an allergist to help determine which vaccine component is responsible for the allergic reaction.

BOX **Abbreviation conventions for influenza vaccines discussed in this report.**

- Main influenza vaccine types:
  - IIV = inactivated influenza vaccine
  - RIV = recombinant influenza vaccine
  - LAIV = live attenuated influenza vaccine
- Numerals following letter abbreviations indicate valency (the number of influenza virus hemagglutinin antigens represented in the vaccine):
  - 3 for trivalent vaccines: one A(H1N1), one A(H3N2), and one B virus (from one lineage)
  - 4 for quadrivalent vaccines: one A(H1N1), one A(H3N2), and two B viruses (one from each lineage)
- All influenza vaccines expected to be available in the United States for the 2024–25 season are trivalent vaccines. However, abbreviations for quadrivalent vaccines (e.g., IIV4) might be used in this report when discussing information specific to quadrivalent vaccines
- Abbreviations for general vaccine categories (e.g., IIV) might be used when discussing information that is not specific to valency or to a specific vaccine in that category.
- Prefixes are used when necessary to refer to certain specific IIVs:
  - a for MF59-adjuvanted inactivated influenza vaccine (e.g., aIIV3)
  - cc for cell culture–based inactivated influenza vaccine (e.g., ccIIV3)
  - HD for high-dose inactivated influenza vaccine (e.g., HD-IIV3)
  - SD for standard-dose inactivated influenza vaccine (e.g., SD-IIV3)

## Methods

ACIP provides annual recommendations for the use of influenza vaccines for the prevention and control of seasonal influenza in the United States. The ACIP Influenza Work Group meets by teleconference once to twice per month throughout the year. Work Group membership includes multiple voting members of ACIP, representatives of ACIP liaison organizations, and consultants. Discussions include topics such as influenza surveillance, vaccine effectiveness and safety, vaccination coverage, program feasibility, cost effectiveness, and vaccine supply. Presentations are requested from invited experts and published and unpublished data are discussed.

The Background Document that supplements this report contains literature related to recommendations made in previous seasons. The information included in the Background Document for such topics is not a systematic review; it is intended to provide an overview of background literature and is periodically updated with literature being identified primarily through a broad search for English-language articles on influenza and influenza vaccines. In general, longstanding recommendations in this document that were made in previous seasons reflect expert opinion, and systematic review and assessment of evidence was not performed. Systematic review and evidence assessment are not performed for minor wording changes to existing recommendations, changes in the Food and Drug Administration (FDA)-recommended viral antigen composition of seasonal influenza vaccines, and minor changes in guidance for the use of influenza vaccines (e.g., guidance for timing of vaccination and other programmatic issues, guidance for dosage in specific populations, guidance for selection of vaccines for specific populations that are already recommended for vaccination, and changes that reflect use that is consistent with FDA-licensed indications and prescribing information).

Typically, systematic review and evaluation of evidence using the Grading of Recommendations Assessment, Development and Evaluation (GRADE) approach (*16*) are performed for new recommendations or substantial changes in the current recommendations (e.g., expansion of the recommendation for influenza vaccination to new populations not previously recommended for vaccination or potential preferential recommendations for specific vaccines).

Evidence is reviewed by the ACIP influenza Work Group, and Work Group considerations are included within the ACIP Evidence to Recommendations framework (EtR) (*17*) to inform the development of recommendations that are proposed for vote by the ACIP. Systematic review, GRADE, and the ACIP EtR framework were used in the development of the updated recommendations for adult solid organ transplant recipients discussed in this report.

## Primary Changes and Updates

Primary changes and updates to the recommendations described in this report include 1) the composition of 2024–25 U.S. seasonal influenza vaccines and 2) updated recommendations for vaccination of adult solid organ transplant recipients. Information relevant to these changes includes the following:

- The composition of the 2024–25 U.S. seasonal influenza vaccines includes an update to the influenza A(H3N2) component. For the 2024–25 season, U.S.-licensed influenza vaccines will contain hemagglutinin (HA) derived from 1) an influenza A/Victoria/4897/2022 (H1N1)pdm09-like virus (for egg-based vaccines) or an influenza A/Wisconsin/67/2022 (H1N1)pdm09-like virus (for cell culture-based and recombinant vaccines, 2) an influenza A/Thailand/8/2022 (H3N2)-like virus (for egg-based vaccines) or an influenza A/Massachusetts/18/2022 (H3N2)-like virus (for cell culture-based and recombinant vaccines), and 3) an influenza B/Austria/1359417/2021 (Victoria lineage)-like virus (for egg-based, cell culture-based, and recombinant vaccines).
- Recommendations for vaccination of adult solid organ transplant recipients have been updated to include HD-IIV3 and aIIV3 as acceptable options for solid organ transplant recipients aged 18 through 64 years who are receiving immunosuppressive medication regimens (without a preference over other age-appropriate IIVs or RIV3). To inform this recommendation, a systematic review Recommendations for the composition of Northern Hemisphere influenza vaccines are made by the World Health Organization (WHO), which organizes a consultation, usually in February of each year. Surveillance data are reviewed, and candidate vaccine viruses are discussed. Information about the WHO meeting of February 2024 for selection of the 2024–25 Northern Hemisphere influenza vaccine composition is available at https://www.who.int/publications/m/item/recommended-composition-of-influenza-virus-vaccines-for-use-in-the-2024-2025-northern-hemisphere-influenza-season. Subsequently, FDA, which has regulatory authority over vaccines in the United States, convenes a meeting of its Vaccines and Related Biological Products Advisory Committee (VRBPAC). This committee considers the recommendations of WHO, reviews and discusses similar data, and makes a final decision regarding the composition of influenza vaccines licensed and marketed in the United States. Materials from the VRBPAC discussion on March 5, 2024, during which the composition of the 2024–25 U.S. influenza vaccines was discussed, are available at https://www.fda.gov/advisory-committees/advisory-committee-calendar/vaccines-and-related-biological-products-advisory-committee-march-5-2024-meeting-announcement. For the 2024–25 influenza season, FDA has recommended that the U.S. seasonal influenza vaccine composition no longer include influenza B/Yamagata, as there have been no confirmed detections of influenza B/Yamagata viruses in global influenza surveillance since March 2020 (*18*,*19*).
  and GRADE of evidence concerning effectiveness and safety of HD-IIV3 and aIIV3 compared with standard-dose unadjuvanted inactivated influenza vaccines was conducted. A summary of this review and the GRADE evidence tables is available at https://www.cdc.gov/vaccines/acip/recs/grade/influenza-solid-organ-transplant.html. A summary of the ACIP EtR framework is available at https://www.cdc.gov/vaccines/acip/recs/grade/influenza-solid-organ-transplant-etr.html.

## Recommendations for the Use of Influenza Vaccines, 2024–25

### Groups Recommended for Vaccination

Routine annual influenza vaccination of all persons aged ≥6 months who do not have contraindications continues to be recommended. All persons should receive an age-appropriate influenza vaccine (one that is approved for their age), with the exception that solid organ transplant recipients aged 18 through 64 years who are receiving immunosuppressive medication regimens may receive either HD-IIV3 or aIIV3 as acceptable options (without a preference over other age-appropriate IIV3s or RIV3) (see Immunocompromised Persons). Influenza vaccines expected to be available for the 2024–25 season, their age indications, and their presentations are described (Table 1). ACIP makes no preferential recommendation for the use of any one influenza vaccine over another when more than one licensed and recommended vaccine is available, except for selection of influenza vaccines for persons aged ≥65 years (see Older Adults). Recommendations regarding timing of vaccination, considerations for specific populations, the use of specific vaccines, and contraindications and precautions are summarized in the sections that follow.

### Timing of Vaccination

Timing of the onset, peak, and decline of influenza activity varies from season to season (*20*). Decisions about timing need to consider the unpredictability of the influenza season, possible waning of vaccine-induced immunity over the course of a season, and practical considerations. For most persons who need only 1 dose of influenza vaccine for the season, vaccination should ideally be offered during September or October. However, vaccination should continue after October and throughout the influenza season as long as influenza viruses are circulating and unexpired vaccine is available. To avoid missed opportunities for vaccination, providers should offer vaccination during routine health care visits and hospitalizations. Revaccination (i.e., providing a booster dose) to persons who have been fully vaccinated for the season is not recommended, regardless of when the current season vaccine was received.

Influenza vaccines might be available as early as July or August; however, vaccination during July and August is not recommended for most groups because of potential waning of immunity over the course of the influenza season (*21*–*40*), particularly among older adults (*21*,*22*,*24*,*31*,*34*,*40*). However, vaccination during July or August can be considered for any recipient for whom there is concern that they will not be vaccinated at a later date. Considerations for timing of vaccination include the following:

- **For most adults (particularly adults aged ≥65 years) and for pregnant persons in the first or second trimester:** Vaccination during July and August should be avoided unless there is concern that vaccination later in the season might not be possible.
- **Children who require 2 doses:** Certain children aged 6 months through 8 years require 2 doses of influenza vaccine for the season (see Children Aged 6 Months Through 8 Years: Number of Influenza Vaccine Doses) (Figure). These children should receive their first dose as soon as possible (including during July and August, if vaccine is available) to allow the second dose (which must be administered ≥4 weeks later) to be received, ideally, by the end of October.

**FIGURE:**
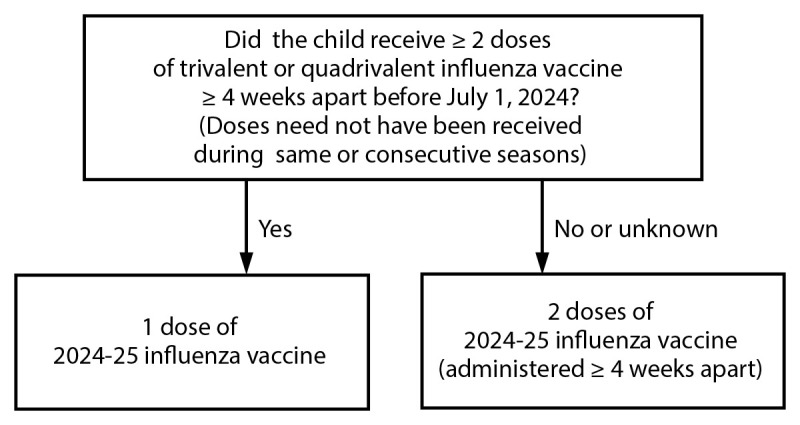
Influenza vaccine dosing algorithm for children aged 6 months through 8 years* — Advisory Committee on Immunization Practices, United States, 2024–25 influenza season. * Children aged 6 months through 8 years who require 2 doses of influenza vaccine should receive their first dose as soon as possible (including during July and August, if vaccine is available) to allow the second dose (which must be administered ≥4 weeks later) to be received, ideally, by the end of October. For children aged 8 years who require 2 doses of vaccine, both doses should be administered even if the child turns age 9 years between receipt of dose 1 and dose 2.
- **Children who require only 1 dose:** Vaccination during July and August can be considered for children of any age who need only 1 dose of influenza vaccine for the season. Although waning of immunity after vaccination over the course of the season has been observed among all age groups (*21*–*40*), there are fewer published studies reporting results specifically among children (*21*,*30*,*32*,*33*,*37*,*39*,*40*). Moreover, children in this group might visit health care providers during the late summer months for medical examinations before the start of school. Vaccination can be considered at this time because it represents a vaccination opportunity.
- **Pregnant persons in the third trimester:** Vaccination during July and August can be considered for pregnant persons who are in the third trimester during these months because vaccination has been associated in multiple studies with reduced risk for influenza illness in their infants during the first months after birth, when they are too young to receive influenza vaccine (*41*–*44*). For pregnant persons in the first or second trimester during July and August, waiting to vaccinate until September or October is preferable, unless there is concern that later vaccination might not be possible.

An increasing number of observational studies (*21*–*40*) have reported decreased vaccine effectiveness with increasing time after vaccination within an influenza season. The rate of waning effectiveness observed in these studies varied considerably and waning effects were inconsistent across age groups, seasons, and influenza virus types and subtypes; although several studies reported faster waning against influenza A(H3N2) viruses than against influenza A(H1N1) or influenza B viruses (*25*,*31*,*35*,*40*). A meta-analysis of 14 studies examining waning of influenza vaccine effectiveness using the test-negative design found a significant decline in effectiveness after vaccination against influenza A(H3N2) and influenza B but not against influenza A(H1N1) (*45*). In that study, VE against influenza A(H3N2) declined, on average, by 32 percentage points, from 45% during the first 3 months to 13% in the fourth to sixth months after vaccination. The rate of waning effectiveness also might vary with age; in several studies, waning was more pronounced among older adults (*21*,*22*,*24*,*31*,*34*,*40*). Several recent multiseason studies of waning protection found that the odds of influenza infection increased by 9% to 28% per month after vaccination among vaccinees of all ages and by 12% to 29% per month among vaccinees aged ≥65 years (*33*,*39*,*40*). There are fewer studies of waning specifically among children, with some reporting waning effectiveness (*21*,*32*,*33*,*37*,*40*) and others finding no evidence of waning effectiveness (*30*,*39*). Complicating the interpretation of studies of waning effectiveness is the fact that observed decreases in protection might be at least partially due to bias, unmeasured confounding, or emergence of antigenic drift variants of influenza viruses that are less well-matched to the vaccine viruses.

Community vaccination programs should balance persistence of vaccine-induced protection through the season with avoiding missed opportunities to vaccinate or vaccinating after onset of influenza circulation occurs. Although delaying vaccination might result in greater immunity later in the season, deferral might result in missed opportunities to vaccinate as well as difficulties in vaccinating a population within a more constrained period. Modeling studies examining the consequences of delaying vaccination (until September or October) among older adults in the United States found that delaying vaccination is beneficial if the delay does not cause a substantial reduction in overall vaccination coverage (because of failure of some persons who would prefer earlier vaccination to get vaccinated later in the fall) (*46*–*48*). Among older adults, delayed vaccination would be beneficial, on balance, if vaccine coverage declines by no more than 6% in a mild season (*47*) or by about 15% in a moderately severe season (*46*,*48*). However, these results are sensitive to many factors, especially the rate of waning of vaccine effectiveness, about which there remains considerable uncertainty.

Vaccination efforts should continue throughout the season because the duration of the influenza season varies, and influenza activity might not occur in certain communities until February, March, or later (*20*). Providers should offer influenza vaccine at health care visits to those not yet vaccinated, and organized vaccination campaigns should continue throughout the influenza season, including after influenza activity has begun in the community. Although vaccination by the end of October is recommended, vaccine administered in December or later, even if influenza activity has already begun, might be beneficial in most influenza seasons. Providers should offer influenza vaccination to unvaccinated persons who have already become ill with influenza during the season because the vaccine might protect them against other circulating influenza viruses.

### Guidance for Influenza Vaccination in Specific Populations and Situations

#### Populations at Higher Risk for Medical Complications Attributable to Severe Influenza

All persons aged ≥6 months who do not have contraindications should be vaccinated annually. However, vaccination to prevent influenza is particularly important for persons who are at increased risk for severe illness and complications from influenza and for influenza-related outpatient, emergency department, or hospital visits. When vaccine supply is limited, vaccination efforts should focus on vaccination of persons at higher risk for medical complications attributable to severe influenza who do not have contraindications. These persons include the following (order of listing does not imply hierarchy or prioritization among these populations):

- All children aged 6 through 59 months.
- All persons aged ≥50 years.
- Adults and children who have chronic pulmonary (including asthma), cardiovascular (excluding isolated hypertension), renal, hepatic, neurologic, hematologic, or metabolic disorders (including diabetes mellitus).
- Persons who are immunocompromised due to any cause (including but not limited to immunosuppression caused by medications or HIV infection).
- Persons who are or will be pregnant during the influenza season.
- Children and adolescents (aged 6 months through 18 years) who are receiving aspirin- or salicylate-containing medications and who might be at risk for experiencing Reye syndrome after influenza virus infection.
- Residents of nursing homes and other long-term care facilities.
- American Indian or Alaska Native persons.
- Persons who are extremely obese (body mass index ≥40 for adults).

IIV3 or RIV3 are suitable for all persons recommended for vaccination, including those in the risk groups listed. LAIV3 is not recommended for certain populations, including certain of these listed groups. Contraindications and precautions for the use of LAIV3 are noted (Table 2).

#### Persons Who Live with or Care for Persons at Higher Risk for Influenza-Related Complications

All persons aged ≥6 months without contraindications should be vaccinated annually. However, emphasis also should be placed on vaccination of persons who live with or care for those who are at increased risk for medical complications attributable to severe influenza. When vaccine supply is limited, vaccination efforts should focus on administering vaccination to persons at higher risk for influenza-related complications as well as persons who live with or care for such persons, including the following:

- Health care personnel, including all paid and unpaid persons working in health care settings who have the potential for exposure to patients or to infectious materials. These personnel might include but are not limited to physicians, nurses, nursing assistants, nurse practitioners, physician assistants, therapists, technicians, emergency medical service personnel, dental personnel, pharmacists, laboratory personnel, autopsy personnel, students and trainees, contractual staff members, and others not directly involved in patient care but who might be exposed to infectious agents (e.g., clerical, dietary, housekeeping, laundry, security, maintenance, administrative, billing staff, and volunteers). ACIP guidance for vaccination of health care personnel has been published previously (*49*).
- Household contacts (including children aged ≥6 months) and caregivers of children aged ≤59 months (<5 years) and adults aged ≥50 years, particularly contacts of children aged <6 months.
- Household contacts (including children aged ≥6 months) and caregivers of persons with medical conditions that put them at higher risk for severe complications from influenza.

Health care personnel and persons who are contacts of persons in these groups (except for of contacts of severely immunocompromised persons who require a protected environment) can receive any influenza vaccine that is otherwise indicated. Persons who care for severely immunocompromised persons requiring a protected environment should not receive LAIV3. ACIP and the Healthcare Infection Control Practices Advisory Committee (HICPAC) have previously recommended that health care personnel who receive LAIV should avoid providing care for severely immunocompromised persons requiring a protected environment for 7 days after vaccination and that hospital visitors who have received LAIV should avoid contact with such persons for 7 days after vaccination (*50*). However, such persons need not be restricted from caring for or visiting less severely immunocompromised persons.

#### Children Aged 6 Through 35 Months: Influenza Vaccine Dose Volumes

Five IIV3s are approved for children aged ≥6 months (Table 1). Four of these vaccines are egg based (Afluria, Fluarix, FluLaval, and Fluzone), and one is cell culture–based (Flucelvax). For these vaccines, the approved dose volumes for children aged 6 through 35 months are as follows (Table 4):

**TABLE 4:** Dose volumes for inactivated influenza vaccines (IIV3s) approved for children aged 6 through 35 months* — United States, 2024–25 influenza season

Trade name (Manufacturer)	Dose volume for children aged 6 through 35 mos (*μ*g HA per vaccine virus)
Afluria (Seqirus)	0.25 mL (7.5 *μ*g)^†^
Fluarix (GlaxoSmithKline)	0.5 mL (15 *μ*g)
Flucelvax (Seqirus)	0.5 mL (15 *μ*g)
FluLaval (GlaxoSmithKline)	0.5 mL (15 *μ*g)
Fluzone (Sanofi Pasteur)	0.5 mL (15 *μ*g)^§^

**Abbreviation:** HA = hemagglutinin.

* For persons aged ≥36 months (≥3 years), the dose volume is 0.5 mL per dose for all inactivated influenza vaccines.

^†^ The approved dose volume for Afluria is 0.25 mL for children aged 6 through 35 months and 0.5 mL for persons aged ≥3 years. However, 0.25-mL prefilled syringes are no longer available. For children aged 6 through 35 months, a 0.25-mL dose must be obtained from a multidose vial.

^§^ Per the package insert, Fluzone is approved for children aged 6 through 35 months at either 0.25 mL or 0.5 mL per dose; however, 0.25-mL prefilled syringes are no longer available. If a prefilled syringe of Fluzone is used for a child in this age group, the dose volume will be 0.5 mL per dose. The 5.0 mL multidose vials should be accessed for only 10 doses, regardless of the volume of the doses obtained or any remaining volume in the vial. Any vaccine remaining in a vial after the maximum number of doses has been removed should be discarded.

- Afluria: 0.25 mL per dose. However, 0.25-mL prefilled syringes are no longer available. For children aged 6 through 35 months, a 0.25-mL dose must be obtained from a multidose vial (*51*).
- Fluarix: 0.5 mL per dose (*52*).
- Flucelvax: 0.5 mL per dose (*53*).
- FluLaval: 0.5 mL per dose (*54*).
- Fluzone: Either 0.25 mL or 0.5 mL per dose. Per the package insert, each dose can be given at either volume (*55*); however, 0.25-mL prefilled syringes are no longer available.

For all of these IIV3s, persons aged ≥36 months (≥3 years) should receive 0.5 mL per dose. Alternatively, healthy children aged ≥24 months (≥2 years) can receive LAIV3, 0.2 mL intranasally (0.1 mL in each nostril) (*56*). LAIV3 is not recommended for certain populations and is not approved for children aged <2 years or adults >49 years (see Contraindications and Precautions for the Use of LAIV3) (Table 2). RIV3 is not approved for children aged <18 years (*57*). High-dose inactivated influenza vaccine (HD-IIV3) (*58*) and adjuvanted inactivated influenza vaccine (aIIV3) (*59*) are not approved for persons aged <65 years.

Care should be taken to administer an age-appropriate vaccine at the appropriate volume for each dose. For IIV3s, the recommended volume can be administered from a prefilled syringe containing the appropriate volume (as supplied by the manufacturer) or a multidose vial. Multidose vials should be used only for the maximum number of doses specified in the package insert. Any vaccine remaining in a vial after the maximum number of doses has been removed should be discarded, regardless of the volume of the doses obtained or any remaining volume in the vial.

#### Children Aged 6 Months Through 8 Years: Number of Influenza Vaccine Doses

Children aged 6 months through 8 years require 2 doses of influenza vaccine administered a minimum of 4 weeks apart during their first season of vaccination for optimal protection (*60*–*63*). Determination of the number of doses needed is based on 1) the child’s age at the time of the first dose of 2024–25 influenza vaccine and 2) the number of doses of influenza vaccine received in previous influenza seasons.

- For children aged 6 months through 8 years, the number of doses of influenza vaccine needed for the 2024–25 influenza season is determined as follows (Figure):
  - Those who have previously received ≥2 total doses of trivalent or quadrivalent influenza vaccine ≥4 weeks apart before July 1, 2024, require only 1 dose for the 2024–25 season. The previous 2 doses of influenza vaccine do not need to have been received in the same season or consecutive seasons.
  - Those who have not previously received ≥2 doses of trivalent or quadrivalent influenza vaccine ≥4 weeks apart before July 1, 2024, or whose previous influenza vaccination history is unknown, require 2 doses for the 2024–25 season. The interval between the 2 doses should be ≥4 weeks. Children aged 6 months through 8 years who require 2 doses of influenza vaccine should receive their first dose as soon as possible (including during July and August, if vaccine is available) to allow the second dose (which must be administered ≥4 weeks later) to be received, ideally, by the end of October. For children aged 8 years who require 2 doses of vaccine, both doses should be administered even if the child turns age 9 years between receipt of dose 1 and dose 2.
- Adults and children aged ≥9 years need only 1 dose of influenza vaccine for the 2024–25 season.

#### Pregnant Persons

Pregnant and postpartum persons are at higher risk for severe illness and complications from influenza, particularly during the second and third trimesters. Influenza vaccination during pregnancy is associated with reduced risk for respiratory illness and influenza among pregnant and postpartum persons as well as infants during the first months of life (*41*–*44*,*64*). ACIP and the American College of Obstetricians and Gynecologists recommend that persons who are pregnant or who might be pregnant or postpartum during the influenza season receive influenza vaccine (*65*). IIV3 or RIV3 can be used. LAIV3 should not be used during pregnancy but can be used postpartum. Influenza vaccine can be administered at any time during pregnancy (i.e., during any trimester), before and during the influenza season. Early vaccination (i.e., during July and August) can be considered for persons who are in the third trimester during these months if vaccine is available because this can provide protection for the infant during the first months of life when they are too young to be vaccinated (*41*–*44*,*64*).

Although experience with the use of IIVs during pregnancy is substantial, data specifically reflecting administration of influenza vaccines during the first trimester are limited. Most studies have not noted an association between influenza vaccination and adverse pregnancy outcomes, including spontaneous abortion (miscarriage) (*66*–*76*). One observational Vaccine Safety Datalink (VSD) study conducted during the 2010–11 and 2011–12 seasons noted an association between receipt of IIV containing influenza A(H1N1)pdm09 and risk for miscarriage in the 28 days after receipt of IIV, when an H1N1pdm09-containing vaccine also had been received the previous season (*77*). However, in a larger VSD follow-up study, IIV was not associated with an increased risk for miscarriage during the 2012–13, 2013–14, and 2014–15 seasons, regardless of previous season vaccination (*78*).

There is less experience with the use of more recently licensed influenza vaccines (e.g., cell culture-based and recombinant vaccines) during pregnancy compared with previously available products. For ccIIV, a review of Vaccine Adverse Event Reporting System (VAERS) reports from 2013 through 2020 (*79*) and a prospective cohort study conducted from 2017 through 2020 (*80*) did not reveal unexpected safety events among pregnant persons. Data from a randomized controlled trial (RCT) conducted at Clinical Immunization Safety Assessment (CISA) Project sites comparing the safety of RIV4 versus IIV4 in 382 pregnant persons supported the safety of RIV4 in pregnancy (https://stacks.cdc.gov/view/cdc/122379) (*81*). Pregnancy registries and surveillance studies exist for certain products, for which information can be found in package inserts.

#### Older Adults

ACIP recommends that adults aged ≥65 years preferentially receive any one of the following higher dose or adjuvanted influenza vaccines: high-dose inactivated influenza vaccine (HD-IIV3), recombinant influenza vaccine (RIV3), or adjuvanted inactivated influenza vaccine (aIIV3). If none of these three vaccines is available at an opportunity for vaccine administration, then any other age-appropriate influenza vaccine should be administered (*82*,*83*).

Older adults (aged ≥65 years) are at increased risk for severe influenza-associated illness, hospitalization, and death compared with younger persons (*4*,*84*,*85*). Influenza vaccines are often less effective in this population (*12*). HD-IIV, RIV, and aIIV have been evaluated in comparison with nonadjuvanted SD-IIVs in this age group. Two of these vaccines, HD-IIV and RIV, are higher dose vaccines, which contain an increased dose of HA antigen per vaccine virus compared with nonadjuvanted SD-IIVs (60 *μ*g for HD-IIV3 and 45 *μ*g for RIV3, compared with 15 *μ*g for standard-dose inactivated vaccines) (*57*,*58*). The adjuvanted vaccine contains 15 *μ*g of HA per virus, similarly to nonadjuvanted SD-IIVs, but contains the adjuvant MF59 (*59*).

HD-IIV, RIV, and aIIV have shown relative benefit compared with SD-IIVs in certain studies, with the most evidence available for HD-IIV3. Randomized efficacy studies comparing these vaccines with nonadjuvanted SD-IIVs against laboratory-confirmed influenza outcomes are few in number (*86*–*88*) and cover few influenza seasons. Observational studies, predominantly retrospective cohort studies using diagnostic code–defined (rather than laboratory-confirmed) influenza outcomes, are more numerous and include more influenza seasons (*89*–*99*). Certain observational studies have reported relative benefit for HD-IIV, RIV, and aIIV in comparison with nonadjuvanted SD-IIVs, particularly in prevention of influenza-associated hospitalizations. The size of this relative benefit has varied from season to season and is not observed in all studies in all seasons, making it difficult to generalize the findings to all or most seasons. Studies directly comparing HD-IIV, RIV, and aIIV with one another are few and do not support a conclusion that any one of these vaccines is consistently superior to the others across seasons (*89*–*91*,*94*,*100*,*101*).

#### Immunocompromised Persons

ACIP recommends that persons with compromised immunity (including but not limited to persons with congenital and acquired immunodeficiency states, persons who are immunocompromised due to medications, and persons with anatomic and functional asplenia) should receive IIV3 or RIV3. All persons should receive an age-appropriate influenza vaccine (i.e., one approved for their age), with the exception that solid organ transplant recipients aged 18 through 64 years who are receiving immunosuppressive medication regimens may receive either HD-IIV3 or aIIV3 as acceptable options (without a preference over other age-appropriate IIV3s or RIV3). ACIP recommends that LAIV3 not be used for immunocompromised persons because of the uncertain but biologically plausible risk for disease attributable to the live vaccine virus. Use of LAIV3 in persons with these and other conditions is discussed in more detail (see Dosage, Administration, Contraindications, and Precautions) (Table 2).

Regarding solid organ transplant recipients specifically, a systematic review and meta-analysis including seven studies pertaining to use of higher dose (HD-IIV, double-dose SD-IIV, and RIV) and MF59-adjuvanted influenza vaccines compared with SD-IIV in this population noted no difference in likelihood of influenza-associated hospitalization (GRADE certainty level Low). However, evidence suggested potentially improved immunogenicity, with greater likelihood of seroconversion for both HD-IIV3 and aIIV3 relative to SD-IIV (GRADE certainty level Moderate for HD-IIV3 vs SD-IIV and Low for aIIV3 vs SD-IIV) for the influenza A(H1N1), influenza A(H3N2), and influenza B vaccine components. There was no evidence of increased risk of graft rejection with either HD-IIV3 or aIIV3 relative to SD-IIV (GRADE certainty level Moderate). Only one study included children. No evidence was available for RIV vs SD-IIV (https://www.cdc.gov/vaccines/acip/recs/grade/influenza-solid-organ-transplant.html; https://www.cdc.gov/vaccines/acip/recs/grade/influenza-solid-organ-transplant-etr.html).

Immunocompromised states comprise a heterogeneous range of conditions with varying risks for severe infections. In many instances, limited data are available regarding the effectiveness of influenza vaccines in the setting of specific immunocompromised states (*102*). Timing of vaccination might be a consideration (e.g., vaccinating during a period either before or after an immunocompromising intervention). The Infectious Diseases Society of America has published detailed guidance for the selection and timing of vaccines for persons with specific immunocompromising conditions (*103*). Immune response to influenza vaccines might be blunted in persons with certain conditions, such as congenital immune deficiencies, and in persons receiving cancer chemotherapy, posttransplant regimens, or immunosuppressive medications.

#### Persons with a History of Guillain-Barré Syndrome After Influenza Vaccination

A history of Guillain-Barré syndrome (GBS) within 6 weeks of a previous dose of any type of influenza vaccine is considered a precaution for influenza vaccination (Table 2). Persons who are not at higher risk for severe influenza complications (see Populations at Higher Risk for Medical Complications Attributable to Severe Influenza) and who are known to have experienced GBS within 6 weeks of a previous influenza vaccination typically should not be vaccinated. As an alternative to vaccination, providers might consider using influenza antiviral chemoprophylaxis for these persons (*104*). However, the benefits of influenza vaccination might outweigh the possible risks for certain persons who have a history of GBS within 6 weeks after receipt of influenza vaccine and who also are at higher risk for severe complications from influenza.

#### Persons with a History of Egg Allergy

ACIP recommends that all persons aged ≥6 months with egg allergy should receive influenza vaccine. Any influenza vaccine (egg based or nonegg based) that is otherwise appropriate for the recipient’s age and health status can be used (https://www.cdc.gov/vaccines/acip/recs/grade/influenza-egg-allergy.html; https://www.cdc.gov/vaccines/acip/recs/grade/influenza-egg-allergy-etr.html). Egg allergy alone necessitates no additional safety measures for influenza vaccination beyond those recommended for any recipient of any vaccine, regardless of severity of previous reaction to egg. All vaccines should be administered in settings in which personnel and equipment needed for rapid recognition and treatment of acute hypersensitivity reactions are available.

Most available influenza vaccines, with the exceptions of RIV3 (Flublok, licensed for persons aged ≥18 years) and ccIIV3 (Flucelvax, licensed for persons aged ≥6 months), are prepared by propagation of virus in embryonated eggs and might contain trace amounts of egg proteins, such as ovalbumin. Among those U.S.-licensed influenza vaccines for which ovalbumin content is reported, quantities are generally small (≤1 *μ*g/0.5mL dose) (*51*,*52*,*54*–*56*,*58*,*59*). Reviews of studies of administration of egg-based influenza vaccines to persons with egg allergy have noted no cases of anaphylaxis or serious hypersensitivity reactions (*105*,*106*). Severe allergic reactions after administration of the egg-free vaccine RIV to egg-allergic persons have been noted in VAERS reports (*107*–*109*). These reports highlight both the possibility that observed reactions after egg-based influenza vaccines might be caused by substances other than egg proteins and the importance of being prepared to recognize and manage serious hypersensitivity reactions when administering any vaccine to any recipient (regardless of allergy history).

Severe and life-threatening reactions to vaccines can rarely occur with any vaccine and in any vaccine recipient, regardless of allergy history. Providers are reminded that all vaccines should be administered in settings in which personnel and equipment needed for rapid recognition and treatment of acute hypersensitivity reactions are available. All vaccination providers should be familiar with their office emergency plan and be certified in cardiopulmonary resuscitation (*110*). No postvaccination observation period is recommended specifically for egg-allergic persons. However, ACIP recommends that vaccination providers consider observing patients (seated or supine) for 15 minutes after administration of any vaccine to decrease the risk for injury should syncope occur (*110*).

Although egg allergy is neither a contraindication nor precaution to the use of any influenza vaccine, there are contraindications and precautions related to allergies to vaccine components other than egg and to previous allergic reactions to influenza vaccines (see Persons with Previous Allergic Reactions to Influenza Vaccines and Dosage, Administration, Contraindications, and Precautions) (Tables 2 and 3).

#### Persons with Previous Allergic Reactions to Influenza Vaccines

As is the case for all vaccines, influenza vaccines contain various components that might cause allergic and anaphylactic reactions. Most influenza vaccine package inserts list among contraindications to their use a history of previous severe allergic reaction (e.g., anaphylaxis) to any component of the vaccine or to a previous dose of any influenza vaccine (*51*,*52*,*54*–*56*,*58*,*59*). For ccIIV3 and RIV3, a history of a severe allergic reaction to any vaccine component is listed as a contraindication; no labeled contraindication is specified for a history of allergic reaction to any other influenza vaccine (*53*,*57*). However, severe allergic reactions, although rare, can occur after influenza vaccination, even among persons with no previous reactions or known allergies. Vaccine components and excipients can be found in package inserts. However, identifying the causative agent without further evaluation (i.e., through evaluation and testing for specific allergies) can be difficult. Severe allergic reactions after vaccination with RIV have been reported to VAERS, certain of which have occurred among persons reporting previous allergic reactions to egg or to influenza vaccines and that might represent a predisposition to allergic manifestations in affected persons (*107*–*109*). Because these rare but severe allergic reactions can occur, ACIP recommends the following for persons with a history of severe allergic reaction to a previous dose of an influenza vaccine (Table 3):

- For egg-based IIV3s and LAIV3:
  - A history of severe allergic reaction (e.g., anaphylaxis) to any influenza vaccine (i.e., any egg-based IIV, ccIIV, RIV, or LAIV of any valency) is a contraindication to future receipt of all egg-based IIV3s and LAIV3. Each individual egg-based IIV3 and LAIV3 is also contraindicated for persons who have had a severe allergic reaction (e.g., anaphylaxis) to any component of that vaccine (excluding egg; see Persons with a History of Egg Allergy).
- For ccIIV3:
  - A history of a severe allergic reaction (e.g., anaphylaxis) to any egg-based IIV, RIV, or LAIV of any valency is a precaution for the use of ccIIV3. If ccIIV3 is administered in such instances, vaccination should occur in an inpatient or outpatient medical setting and should be supervised by a health care provider who is able to recognize and manage severe allergic reactions. Providers also can consider consultation with an allergist to help determine the vaccine component responsible for the allergic reaction.
  - A history of a severe allergic reaction (e.g., anaphylaxis) to any ccIIV of any valency or to any component of ccIIV3 is a contraindication to future receipt of ccIIV3.
- For RIV3:
  - A history of a severe allergic reaction (e.g., anaphylaxis) to any egg-based IIV, ccIIV, or LAIV of any valency is a precaution for the use of RIV3. If RIV3 is administered in such instances, vaccination should occur in an inpatient or outpatient medical setting and should be supervised by a health care provider who is able to recognize and manage severe allergic reactions. Providers can also consider consultation with an allergist to help determine the vaccine component responsible for the allergic reaction.
  - A history of a severe allergic reaction (e.g., anaphylaxis) to any RIV of any valency or to any component of RIV3 is a contraindication to future receipt of RIV3.

#### Vaccination Issues for Travelers

In temperate climate regions of the Northern and Southern Hemispheres, influenza activity is seasonal, occurring during approximately October–May in the Northern Hemisphere and April–September in the Southern Hemisphere. In the tropics, influenza might occur throughout the year (*111*). The timing of influenza activity and predominant types and subtypes of influenza viruses in circulation vary by geographic region (*112*). Travelers can be exposed to influenza when traveling to an area where influenza is circulating or when traveling as part of large tourist groups (e.g., on cruise ships) that include persons from areas of the world where influenza viruses are circulating (*113*–*116*).

Travelers who want to reduce their risk for influenza should consider influenza vaccination, preferably at least 2 weeks before departure. In particular, persons who live in the United States and are at higher risk for influenza complications and who were not vaccinated with influenza vaccine during the previous Northern Hemisphere fall or winter should consider receiving influenza vaccination before departure if they plan to travel to the tropics, to the Southern Hemisphere during the Southern Hemisphere influenza season (April–September), or with organized tourist groups or on cruise ships to any location. Persons at higher risk who received the previous season’s influenza vaccine before travel should consult with their health care provider to discuss the risk for influenza and other travel-related diseases before embarking on travel during the summer. All persons (regardless of risk status) who are vaccinated in preparation for travel before the upcoming influenza season’s vaccine is available, or who received the immediately preceding Southern Hemisphere influenza vaccine, should receive the current U.S. seasonal influenza vaccine the following fall or winter.

Influenza vaccine formulated for the Southern Hemisphere might differ in viral composition from the Northern Hemisphere vaccine. For persons traveling to the Southern Hemisphere during the Southern Hemisphere influenza season, receipt of a current U.S.-licensed Southern Hemisphere influenza vaccine formulation before departure might be reasonable but might not be feasible because of limited access to or unavailability of Southern Hemisphere formulations in the United States. Most Southern Hemisphere influenza vaccine formulations are not licensed in the United States, and they are typically not commercially available. More information on influenza vaccines and travel is available at https://wwwnc.cdc.gov/travel/diseases/influenza-seasonal-zoonotic-and-pandemic. Additional information on global influenza surveillance by region is available at https://www.who.int/tools/flunet.

#### Use of Influenza Antiviral Medications

Administration of any IIV3 or RIV3 to persons receiving influenza antiviral medications for treatment or chemoprophylaxis of influenza is acceptable. Data concerning vaccination with LAIV3 in the setting of influenza antiviral use are not available. However, influenza antiviral medications might interfere with the action of LAIV3 because this vaccine contains live influenza viruses.

The package insert for LAIV3 notes that influenza antiviral agents might reduce the effectiveness of the vaccine if administered within the interval from 48 hours before to 14 days after vaccination (*56*). However, the newer influenza antivirals peramivir and baloxavir have longer half-lives than oseltamivir and zanamivir, approximately 20 hours for peramivir (*117*) and 79 hours for baloxavir (*118*), and could potentially interfere with the replication of LAIV3, if administered >48 hours before vaccination. Potential interactions between influenza antivirals and LAIV3 have not been studied, and the ideal intervals between administration of these medications and LAIV3 are not known. Assuming a period of at least 5 half-lives for substantial decrease in drug levels (*119*), a reasonable assumption is that peramivir might interfere with the mechanism of LAIV3 if administered from 5 days before through 2 weeks after vaccination and baloxavir might interfere if administered from 17 days before through 2 weeks after vaccination. The interval between influenza antiviral receipt and LAIV3 during which interference might occur could be further prolonged in the presence of medical conditions that delay medication clearance (e.g., renal insufficiency). Persons who receive these medications during these periods before or after receipt of LAIV3 should be revaccinated with another appropriate influenza vaccine (e.g., IIV3 or RIV3).

#### Administration of Influenza Vaccines with Other Vaccines

IIV3s and RIV3 can be administered simultaneously or sequentially with other inactivated vaccines or live vaccines. Injectable vaccines that are given concomitantly should be administered at separate anatomic sites. Vaccines that are administered at the same time as influenza vaccines that might be more likely to be associated with local injection site reactions (e.g., HD-IIV3 and aIIV3) should be given in different limbs, if possible. LAIV3 can be administered simultaneously with other live or inactivated vaccines. However, if two live vaccines are not given simultaneously, at least 4 weeks should pass after administration of one live vaccine (such as LAIV3) before another live vaccine is administered (*110*).

In recent years, multiple vaccines containing nonaluminum adjuvants have been licensed for use in the United States for the prevention of various infectious diseases. Examples include AS01_B_ (in Shingrix, recombinant zoster subunit vaccine [RZV]) (*120*), AS01_E_ (in Arexvy, respiratory syncytial virus vaccine) (*121*) MF59 (in Fluad [aIIV3]) (*59*), and cytosine phosphoguanine oligodeoxynucleotide (in Heplisav-B, recombinant hepatitis B surface antigen vaccine) (*122*). Data are limited regarding coadministration of these vaccines with other adjuvanted or nonadjuvanted vaccines, including COVID-19 vaccines. Coadministration of RZV with nonadjuvanted IIV4 has been studied, and no evidence of decreased immunogenicity or safety concerns was noted (*123*). A CISA RCT in persons aged ≥65 years found that the proportion of participants with at least one severe local or systemic reaction was not higher after simultaneous administration of RZV dose 1 and quadrivalent adjuvanted inactivated influenza vaccine compared with simultaneous administration of RZV dose 1 and quadrivalent high-dose inactivated influenza vaccine (*124*). Data on the immunogenicity and safety of simultaneous or sequential administration of two nonaluminum adjuvant–containing vaccines are limited, and the ideal interval between such vaccines when given sequentially is not known. In the study of Shingrix and nonadjuvanted IIV4 (*123*), most reactogenicity symptoms resolved within 4 days. Because of the limited data on the safety of simultaneous administration of two or more vaccines containing nonaluminum adjuvants and the availability of nonadjuvanted influenza vaccine options, selection of a nonadjuvanted influenza vaccine can be considered in situations in which influenza vaccine and another vaccine containing a nonaluminum adjuvant are to be administered concomitantly. However, influenza vaccination should not be delayed if a specific vaccine is not available. As recommended for all vaccines, vaccines with nonaluminum adjuvants should be administered at separate anatomic sites from other vaccines that are given concomitantly (*110*).

For more recently introduced and new vaccines, data informing simultaneous administration with influenza vaccines might be limited or evolving. Providers should consult current CDC/ACIP recommendations and guidance for up-to-date information.

## Influenza Vaccine Composition and Available Vaccines

### Influenza Vaccine Composition for the 2024–25 Season

All influenza vaccines licensed in the United States will contain components derived from influenza viruses antigenically similar to those recommended by FDA (https://www.fda.gov/advisory-committees/advisory-committee-calendar/vaccines-and-related-biological-products-advisory-committee-march-5-2024-meeting-announcement) (*125*). All influenza vaccines expected to be available in the United States for the 2024–25 season will be trivalent vaccines. For the 2024–25 season, U.S. egg-based influenza vaccines (i.e., vaccines other than ccIIV3 and RIV3) will contain HA derived from

- an influenza A/Victoria/4897/2022 (H1N1)pdm09-like virus,
- an influenza A/Thailand/8/2022 (H3N2)-like virus, and
- an influenza B/Austria/1359417/2021 (Victoria lineage)-like virus.

For the 2024–25 season, U.S. cell culture–based inactivated (ccIIV3) and recombinant (RIV3) influenza vaccines will contain HA derived from

- an influenza A/Wisconsin/67/2022 (H1N1)pdm09-like virus,
- an influenza A/Massachusetts/18/2022 (H3N2)-like virus, and
- an influenza B/Austria/1359417/2021 (Victoria lineage)-like virus

### Vaccines Available for the 2024–25 Season

Availability of specific types and brands of licensed seasonal influenza vaccines in the United States is determined by the manufacturers of the vaccines. Information presented concerning vaccines expected to be available and their approved indications and usage reflects current knowledge and is subject to change.

Various influenza vaccines will be available for the 2024–25 season (Table 1). For many vaccine recipients, more than one type or brand of vaccine might be appropriate within approved indications and ACIP recommendations. Current prescribing information and ACIP recommendations should be consulted for up-to-date information. Contraindications and precautions for the different types of influenza vaccines are summarized (Tables 2 and 3), as are dose volumes (Table 4).

Not all influenza vaccines are likely to be uniformly available in any specific practice setting or geographic locality. Vaccination should not be delayed to obtain a specific product when an appropriate one is available. Within these guidelines and approved indications, ACIP makes no preferential recommendation for the use of any one influenza vaccine over another when more than one licensed and recommended vaccine is available, except for selection of influenza vaccines for persons aged ≥65 years (see Older Adults).

### Dosage, Administration, Contraindications, and Precautions

#### Trivalent Inactivated Influenza Vaccines (IIV3s)

**Available Vaccines.** As in recent seasons, various inactivated influenza vaccines (IIVs) are expected to be available for 2024–25 (Table 1); all are expected to be trivalent (IIV3s). Standard-dose, nonadjuvanted IIV3s are licensed for persons aged as young as 6 months. However, for certain IIV3s, the approved dose volume for children aged 6 through 35 months differs from that for older children and adults (Table 4). Care should be taken to administer the appropriate dose volume. Two IIV3s, the MF59-adjuvanted IIV3 Fluad (aIIV3) and the high-dose IIV3 Fluzone High-Dose (HD-IIV3), are approved only for persons aged ≥65 years, but are acceptable options for solid organ transplant recipients aged 18 through 64 years who are receiving immunosuppressive medication regimens, without a preference over other age-appropriate IIV3s or RIV3.

Standard-dose, nonadjuvanted IIV3s contain 15 *μ*g of HA per vaccine virus in a 0.5-mL dose (7.5 *μ*g of HA per vaccine virus in a 0.25-mL dose). For 2024–25, this category is expected to include five different vaccines (Table 1). Four of these are egg-based vaccines (Afluria, Fluarix, FluLaval, and Fluzone), and one is a cell culture–based vaccine (Flucelvax [ccIIV3]). All are approved for persons aged ≥6 months. Egg-based and cell culture–based vaccines differ in the substrate in which reference vaccine viruses supplied to the manufacturer are propagated in quantities sufficient to produce the needed number of doses of vaccine. For the IIV3s Afluria (*51*), Fluarix (*52*), FluLaval (*54*), and Fluzone (*55*), reference vaccine viruses are propagated in eggs. For Flucelvax (ccIIV3), reference vaccine viruses are propagated in Madin-Darby canine kidney cells instead of eggs (*53*).

Two additional IIV3s that will be available for the 2024–25 season are approved only for persons aged ≥65 years. These vaccines are egg based. Trivalent high-dose inactivated influenza vaccine (Fluzone High-Dose; HD-IIV3) contains 60 *μ*g of HA per vaccine virus (180 *μ*g total) in a 0.5-mL dose (*58*). Trivalent adjuvanted inactivated influenza vaccine (Fluad; aIIV3) contains 15 *μ*g of HA per vaccine virus (45 *μ*g total) and MF59 adjuvant (*59*).

**Dosage and Administration.** Standard-dose nonadjuvanted IIV3s are approved for children aged as young as 6 months. Certain of these IIV3s are approved at different dose volumes for very young children than for older children and adults. Care should be taken to administer the correct dose volume for each needed dose (see Children Aged 6 Through 35 Months: Influenza Vaccine Dose Volumes) (Tables 1 and 4):

- Afluria: The approved dose volume for children aged 6 through 35 months is 0.25 mL per dose. Persons aged ≥36 months (≥3 years) should receive 0.5 mL per dose (*51*).
- Fluarix: The approved dose volume is 0.5 mL per dose for all persons aged ≥6 months (*52*).
- Flucelvax: The approved dose volume is 0.5 mL per dose for all persons aged ≥6 months (*53*).
- FluLaval: The approved dose volume is 0.5 mL per dose for all persons aged ≥6 months (*54*).
- Fluzone: The approved dose volume for children aged 6 through 35 months is either 0.25 mL or 0.5 mL per dose. Persons aged ≥36 months (≥3 years) should receive 0.5 mL per dose (*55*).

If prefilled syringes are not available, the appropriate volume can be administered from a multidose vial. Of note, dose volume is distinct from the number of doses. Children in this age group who require 2 doses for 2024–25 need 2 separate doses administered ≥4 weeks apart, regardless of the specific IIV3 used and volume given for each dose (see Children Aged 6 Months Through 8 Years: Number of Influenza Vaccine Doses) (Figure).

For children aged 36 months (3 years) through 17 years and adults aged ≥18 years, the dose volume for all IIV3s is 0.5 mL per dose. If a smaller vaccine dose (e.g., 0.25 mL) is inadvertently administered to a person aged ≥36 months, the remaining volume needed to make a full dose should be administered during the same vaccination visit or, if measuring the needed remaining volume is a challenge, administering a repeat dose at the full volume is acceptable. If the error is discovered later (after the recipient has left the vaccination setting), a full dose should be administered as soon as the recipient can return. Vaccination with a formulation approved for adult use should be counted as a single dose if inadvertently administered to a child.

IIV3s are administered intramuscularly (IM). For adults and older children, the deltoid muscle is the preferred site. Infants and younger children should be vaccinated in the anterolateral thigh. Additional specific guidance regarding site selection and needle length for IM injection is provided in the General Best Practice Guidelines for Immunization (*110*). One IIV3, Afluria, is licensed for IM injection via the PharmaJet Stratis jet injector for persons aged 18 through 64 years (*51*). Persons in this age group can receive Afluria via either needle and syringe or this specific jet injection device. Children aged 6 months through 17 years and adults aged ≥65 years should receive this vaccine by needle and syringe only. No other IIV3s are licensed for administration by jet injector.

**Contraindications and Precautions for the Use of IIV3s.** Manufacturer package inserts and updated CDC and ACIP guidance should be consulted for information on contraindications and precautions for individual influenza vaccines. Each IIV3, whether egg based or cell culture based, has a labeled contraindication for persons with a history of a severe allergic reaction to any component of that vaccine (Tables 2 and 3). However, although egg is a component of all IIV3s other than ccIIV3, ACIP makes specific recommendations for the use of influenza vaccine for persons with egg allergy (see Persons with a History of Egg Allergy). All egg-based IIV3s are contraindicated in persons who have had a severe allergic reaction (e.g., anaphylaxis) to a previous dose of any influenza vaccine (any egg-based IIV, ccIIV, RIV, or LAIV of any valency). Use of ccIIV3 is contraindicated in persons who have had a severe allergic reaction (e.g., anaphylaxis) to any ccIIV of any valency. A history of severe allergic reaction (e.g., anaphylaxis) to any other influenza vaccine (i.e., any egg-based IIV, RIV, or LAIV of any valency) is a precaution for the use of ccIIV3 (see Persons with Previous Allergic Reactions to Influenza Vaccines) (Tables 2 and 3). If ccIIV3 is administered in such an instance, vaccination should occur in an inpatient or outpatient medical setting and should be supervised by a health care provider who is able to recognize and manage severe allergic reactions. Providers can also consider consultation with an allergist to help identify the vaccine component responsible for the reaction. Information about vaccine components can be found in the package inserts for each vaccine. Prophylactic use of antiviral agents is an option that can be considered for preventing influenza among persons who cannot receive vaccine, particularly for those who are at higher risk for medical complications attributable to severe influenza (*104*).

Moderate or severe acute illness with or without fever is a general precaution for vaccination (*110*). A history of GBS within 6 weeks after receipt of a previous dose of influenza vaccine is considered a precaution for the use of all influenza vaccines (Table 2).

#### Trivalent Recombinant Influenza Vaccine (RIV3)

**Available Vaccine.** One recombinant influenza vaccine, Flublok (RIV3), is expected to be available during the 2024–25 influenza season. RIV3 is approved for persons aged ≥18 years. This vaccine contains recombinant HA produced in an insect cell line using genetic sequences from cell-derived influenza viruses and is manufactured without the use of influenza viruses or eggs (*57*).

**Dosage and Administration**. RIV3 is administered by IM injection via needle and syringe. A 0.5-mL dose contains 45 *μ*g of HA derived from each vaccine virus (135 *μ*g total).

**Contraindications and Precautions for the Use of RIV3.** Manufacturer package inserts and updated CDC and ACIP guidance should be consulted for information on contraindications and precautions for individual influenza vaccines. RIV3 is contraindicated in persons who have had a severe allergic reaction (e.g., anaphylaxis) to a previous dose of any RIV of any valency or to any component of RIV3. A history of a severe allergic reaction (e.g., anaphylaxis) to any other influenza vaccine (i.e., any egg-based IIV, ccIIV, or LAIV of any valency) is a precaution for the use of RIV3. If RIV3 is administered in such an instance, vaccination should occur in an inpatient or outpatient medical setting and should be supervised by a health care provider who is able to recognize and manage severe allergic reactions. Providers can also consider consulting with an allergist to help identify the vaccine component responsible for the reaction (Tables 2 and 3).

Moderate or severe acute illness with or without fever is a general precaution for vaccination (*110*). A history of GBS within 6 weeks after receipt of a previous dose of influenza vaccine is considered a precaution for the use of all influenza vaccines (Table 2). RIV3 is not approved for children aged <18 years.

#### Trivalent Live Attenuated Influenza Vaccine (LAIV3)

**Available Vaccine.** One live attenuated influenza vaccine, FluMist (LAIV3), is expected to be available during the 2024–25 influenza season. LAIV3 is approved for persons aged 2 through 49 years. LAIV3 contains live attenuated influenza viruses that are propagated in eggs. These viruses are cold adapted (so that they replicate efficiently at 25°C [77°F]) and temperature sensitive (so that their replication is restricted at higher temperatures, 39°C [102.2°F] for influenza A viruses and 37°C [98.6°] for influenza B viruses). The live attenuated vaccine viruses replicate in the nasopharynx, which is necessary to promote an immune response (*56*). No preference is expressed for LAIV3 versus other influenza vaccines used within specified indications.

**Dosage and Administration.** LAIV3 is administered intranasally using the supplied prefilled, single-use sprayer containing 0.2 mL of vaccine. Approximately 0.1 mL (i.e., one half of the total sprayer contents) is sprayed into the first nostril while the recipient is in the upright position. An attached dose-divider clip is removed from the sprayer to permit administration of the second half of the dose into the other nostril. Sniffing of the dose is not necessary. If the recipient sneezes immediately after administration, the dose should not be repeated. However, if nasal congestion is present that might impede delivery of the vaccine to the nasopharyngeal mucosa, deferral of administration should be considered until resolution of the illness, or another appropriate vaccine should be administered instead. Each total dose of 0.2 mL contains 10^6.5–7.5^ fluorescent focus units of each vaccine virus (*56*).

**Contraindications and Precautions for the Use of LAIV3.** Manufacturer package inserts and updated CDC and ACIP guidance should be consulted for information on contraindications and precautions for individual influenza vaccines. Conditions considered by ACIP to be contraindications and precautions for the use of LAIV3 are summarized (Table 2). These include two labeled contraindications that appear in the package insert (*56*) and other conditions for which there is either uncertain but biologically plausible potential risk associated with live viruses or limited data for use of LAIV. Contraindications to use of LAIV3 include the following (Tables 2 and 3):

- Severe allergic reaction (e.g., anaphylaxis) to any component of the vaccine or to a previous dose of any influenza vaccine (i.e., any egg-based IIV, ccIIV, RIV, or LAIV of any valency; a labeled contraindication noted in the package insert). However, although egg is a component of LAIV3, ACIP makes specific recommendations for the use of influenza vaccine for persons with egg allergy (see Persons with a History of Egg Allergy).
- Children and adolescents receiving concomitant aspirin- or salicylate-containing medications, because of the potential risk for Reye syndrome (a labeled contraindication noted in the package insert).
- Children aged 2 through 4 years who have received a diagnosis of asthma or whose parents or caregivers report that a health care provider has told them during the preceding 12 months that their child had wheezing or asthma or whose medical record indicates a wheezing episode has occurred during the preceding 12 months.
- Children and adults who are immunocompromised due to any cause, including but not limited to immunosuppression caused by medications, congenital or acquired immunodeficiency states, HIV infection, anatomic asplenia, or functional asplenia (such as that due to sickle cell anemia).
- Close contacts and caregivers of severely immunosuppressed persons who require a protected environment.
- Pregnancy.
- Persons with active communication between the cerebrospinal fluid (CSF) and the oropharynx, nasopharynx, nose, or ear or any other cranial CSF leak.
- Persons with cochlear implants, because of the potential for CSF leak that might exist for a period after implantation (providers might consider consultation with a specialist concerning the risk for persistent CSF leak if an inactivated or recombinant vaccine cannot be used).
- Receipt of influenza antiviral medication within the previous 48 hours for oseltamivir and zanamivir, previous 5 days for peramivir, and previous 17 days for baloxavir. The interval between influenza antiviral receipt and LAIV3 during which interference might potentially occur might be further prolonged in the presence of medical conditions that delay medication clearance (e.g., renal insufficiency).

Precautions to the use of LAIV3 include the following (Tables 2 and 3):

- Moderate or severe acute illness with or without fever.
- History of GBS within 6 weeks after receipt of any influenza vaccine.
- Asthma in persons aged ≥5 years.
- Other underlying medical condition (other than those listed under contraindications) that might predispose to complications after wild-type influenza virus infection (e.g., chronic pulmonary, cardiovascular [except isolated hypertension], renal, hepatic, neurologic, hematologic, or metabolic disorders [including diabetes mellitus]).

## Storage and Handling of Influenza Vaccines

In all instances, approved manufacturer packaging information should be consulted for authoritative guidance concerning storage and handling of specific influenza vaccines. Typically, influenza vaccines should be protected from light and stored at temperatures that are recommended in the package insert. Recommended storage temperatures are typically 36°F–46°F (2°C–8°C) and should be maintained at all times with adequate refrigeration and temperature monitoring. Vaccine that has frozen should be discarded. Specific recommendations for appropriate refrigerators and temperature monitoring equipment can be found in the Vaccine Storage and Handling Toolkit, available at https://www.cdc.gov/vaccines/hcp/storage-handling/?CDC_AAref_Val=https://www.cdc.gov/vaccines/hcp/admin/storage/toolkit/index.html.

Vaccines should not be used beyond the expiration date on the label. In addition to the expiration date, multidose vials also might have a beyond-use date (BUD), which specifies the number of days the vaccine can be kept once first accessed. After being accessed for the first dose, multidose vials should not be used after the BUD. If no BUD is provided, then the listed expiration date is to be used. Multidose vials should be returned to recommended storage conditions between uses. Package information might also specify a maximum number of doses contained in multidose vials (regardless of remaining volume). No more than the specified number of doses should be removed, and any remainder should be discarded. Providers should contact the manufacturer for information on permissible temperature excursions and other departures from recommended storage and handling conditions that are not discussed in the package labeling.

## Additional Sources of Information Regarding Influenza and Influenza Vaccines

### Influenza Surveillance, Prevention, and Control

Updated information regarding influenza surveillance, detection, prevention, and control is available at https://www.cdc.gov/flu. U.S. surveillance data are updated weekly throughout the year on FluView (https://www.cdc.gov/flu/weekly) and can be viewed in FluView Interactive (https://www.cdc.gov/flu/weekly/fluviewinteractive.htm). In addition, periodic updates regarding influenza are published in MMWR (https://www.cdc.gov/mmwr/index.html). Additional information regarding influenza and influenza vaccines can be obtained from CDCINFO by calling 1–800–232–4636. State and local health departments should be consulted about availability of influenza vaccines, access to vaccination programs, information related to state or local influenza activity, reporting of influenza outbreaks and influenza-related pediatric deaths, and advice concerning outbreak control.

### Vaccine Adverse Event Reporting System (VAERS)

The National Childhood Vaccine Injury Act of 1986 requires health care providers to report any adverse event listed by the vaccine manufacturer as a contraindication to future doses of the vaccine or any adverse event listed in the VAERS Table of Reportable Events Following Vaccination (https://vaers.hhs.gov/docs/VAERS_Table_of_Reportable_Events_Following_Vaccination.pdf) that occurs within the specified period after vaccination. In addition to mandated reporting, health care providers are encouraged to report any clinically significant adverse event after vaccination to VAERS. Information on how to report a vaccine adverse event is available at https://vaers.hhs.gov/index.html.

### National Vaccine Injury Compensation Program (VICP)

The National Vaccine Injury Compensation Program (VICP), established by the National Childhood Vaccine Injury Act of 1986, as amended, is a no-fault alternative to the traditional tort system. It provides compensation to persons found to be injured by certain vaccines. VICP covers most vaccines routinely given in the United States. The Vaccine Injury Table (https://www.hrsa.gov/sites/default/files/hrsa/vicp/vaccine-injury-table-01-03-2022.pdf) lists the vaccines covered by VICP and the associated injuries and conditions that might receive a legal presumption of causation. If the injury or condition is not in the table or does not meet the requirements in the table, persons must prove that the vaccine caused the injury or condition. Claims must be filed within specified time frames. Persons of all ages who receive a VICP-covered vaccine might be eligible to file a claim. Additional information is available at https://www.hrsa.gov/vaccine-compensation or by calling 1–800–338–2382.

### Additional Resources

#### ACIP Statements

- Recommended Adult Immunization Schedule for Ages 19 Years or Older, United States: https://www.cdc.gov/vaccines/hcp/imz-schedules/adult-age.html?CDC_AAref_Val=https://www.cdc.gov/vaccines/schedules/hcp/imz/adult.html
- Recommended Child and Adolescent Immunization Schedule for Ages 18 Years or Younger, United States: https://www.cdc.gov/vaccines/schedules/hcp/imz/child-adolescent.html
- Immunization of Health Care Personnel: Recommendations of the Advisory Committee on Immunization Practices (ACIP), 2011. MMWR Recomm Rep 2011;60(No.RR-7):1–45: https://www.cdc.gov/mmwr/preview/mmwrhtml/rr6007a1.htm

#### General Best Practice Guidelines for Immunization:

- General Best Practice Guidelines for Immunization: https://www.cdc.gov/vaccines/hcp/acip-recs/general-recs/index.html

#### COVID-19 Vaccine Recommendations and Guidance

- ACIP recommendations for the use of COVID-19 vaccines: https://www.cdc.gov/acip-recs/hcp/vaccine-specific/covid-19.html?CDC_AAref_Val=https://www.cdc.gov/vaccines/hcp/acip-recs/vacc-specific/covid-19.html
- Clinical Care Considerations for COVID-19 Vaccination: https://www.cdc.gov/vaccines/covid-19/clinical-considerations/index.html
- Use of COVID-19 Vaccines in the United States—Interim Clinical Considerations: https://www.cdc.gov/vaccines/covid-19/clinical-considerations/covid-19-vaccines-us.html
- FDA COVID-19 Vaccines page: https://www.fda.gov/emergency-preparedness-and-response/coronavirus-disease-2019-covid-19/covid-19-vaccines

#### Vaccine Information Sheets

- IIV3 and RIV3: https://www.cdc.gov/vaccines/hcp/vis/vis-statements/flu.pdf
- LAIV3: https://www.cdc.gov/vaccines/hcp/vis/vis-statements/flulive.pdf

#### Influenza Vaccine Package Inserts

- https://www.fda.gov/vaccines-blood-biologics/vaccines/vaccines-licensed-use-united-states

#### CDC Influenza Antiviral Guidance

- Influenza Antiviral Medications: Summary for Clinicians: https://www.cdc.gov/flu/professionals/antivirals/summary-clinicians.htm

#### Infectious Diseases Society of America Influenza Antiviral Guidance

- Clinical Practice Guidelines by the Infectious Diseases Society of America: 2018 Update on Diagnosis, Treatment, Chemoprophylaxis, and Institutional Outbreak Management of Seasonal Influenza: https://academic.oup.com/cid/article/68/6/e1/5251935
- American Academy of Pediatrics Guidance
- American Academy of Pediatrics Recommendations for Prevention and Control of Influenza in Children (Red Book Online): https://publications.aap.org/redbook

#### Infectious Diseases Society of America Guidance for Vaccination of Immunocompromised Hosts

- 2013 IDSA Clinical Practice Guideline for Vaccination of the Immunocompromised Host: https://academic.oup.com/cid/article/58/3/e44/336537

#### American College of Obstetricians and Gynecologists

- Influenza in Pregnancy: Prevention and Treatment: https://www.acog.org/clinical/clinical-guidance/committee-statement/articles/2024/02/influenza-in-pregnancy-prevention-and-treatment
